# Harm Reduction and Moral Desert in the Context of Drug Policy

**DOI:** 10.1007/s10728-020-00411-z

**Published:** 2020-10-27

**Authors:** Lindsey Brooke Porter

**Affiliations:** Centre for Health, Law and Society, University of Bristol Law School, 8-10 Berkeley Square, Bristol, BS8 1HH UK

**Keywords:** Drug policy, Harm reduction, Moral desert, Avoidable harm

## Abstract

The target of my discussion is intuitions lay people have about justice in the context of drug policy—intuitions that take on a more or less moral-desert-based shape. I argue that even if we think desert is the right measure of how we ought to treat people, we ought still be in favour of Harm Reduction measures for people who use drugs. Harm Reduction measures are controversial with members of the public, and much of the opposition seems to come from something like an appeal to a desert conception of justice—the notion that a just state of affairs is one in which everybody gets what they deserve, no more, no less. A recent study, for example, found that ‘moral outrage’ predicts a preference for prevalence reduction (criminal sanction, etc.) over Harm Reduction. The thinking seems to be that, since drug use is wrong, letting people who use drugs suffer and/or die as a consequence of their use is just. Aiding their health and safety, while perhaps compassionate, is unjust. I argue that there is a bad desert fit between using drugs and suffering avoidable harm even if using drugs is morally wrong. Many of the possible harms of drug use are socially/policy driven, and much problematic drug use is context dependent, not cleanly attributable to the decisions of the person who uses drugs. This means that even if drug use is wrong, people who use drugs deserve Harm Reduction policies, at minimum.

## Introduction

Much of the opposition to Harm Reduction approaches to drug policy seems to come from something like an appeal to a particular conception of justice: what’s usually called ‘justice as desert’. On this account of the nature of justice, as Rawls put it, justice is *happiness according to virtue* [10]. What is just is that people get what they deserve: happiness and wellbeing if they or their own actions are morally virtuous, or unhappiness if they/their actions are vicious.^1^

With respect to the apparent vice of drug use, justice as desert might have it that people who use drugs (PWUD) don’t deserve for public funds to be spent on—or policy to be altered towards the end of—reducing the harms they suffer as a result of drug use because *they were (morally) wrong to use drugs in the first place*. The thinking, then, is that since PWUD create and maintain the circumstances that put them in harm’s way via their own bad actions, we (we society, we taxpayers, we health professionals and other service providers) are under no obligation to aid them in avoiding harm. More so, justice demands that we *not* do so.

Even many advocates of Harm Reduction approaches to drug policy (dHR) tacitly assume that in order to support Harm Reduction measures, one must set aside questions of moral desert (e.g. [1, 4, 7, 14]). In other words, even many proponents of dHR tacitly agree that dHR may be incompatible with what PWUD deserve.

In this paper, I will argue that even on a desert conception of justice, justice demands harm reduction. My aim is not to defend such a conception of justice: it is unclear whether this conception of justice, or indeed of morality, is one we should accept. Rather, I will argue that *even if* we think desert is the right measure of how we ought to treat people, we ought still be in favour of dHR. In section one of the paper, I will discuss harm reduction, as a policy approach and a locus of division. In section two, I will give a brief synopsis of desert conceptions of justice. In section three, I will explore why we might think that justice as desert demands a prohibitionist, or at least a non-dHR approach, and argue that this thinking is mistaken: justice—even justice as desert—demands harm reduction.

## Harm Reduction

Needle exchange programmes are usually taken to be the paradigm example of harm reduction. Other harm reduction measures include, inter alia, supervised injection sites, STI testing for sex workers and PWUD, comprehensive sex education for teens, and—depending who you ask—things like seat belt laws, e-cigarettes and cannabis legalisation.

The phrase “harm reduction” means slightly different things to different people—and to much of the public, it means nothing at all. For the purposes of this paper, I will be conceptualising harm reduction in the drugs context as

policies, measures, or approaches that seek to reduce the physical, mental, and/or social harms of a behaviour without thereby seeking to reduce the prevalence of such behaviour.^2^

Harm reduction—especially dHR—is controversial in many quarters and jurisdictions, as well as with many members of the public. There is, furthermore, empirical evidence that ‘moral outrage’ predicts a preference for prevalence reduction over harm reduction [8]. That is, people who take strong moral issue with drug use are more likely to strongly oppose dHR, presumably on grounds that PWUD don’t deserve protection from the consequences of their use [12].

In the context of drug policy, one unavoidable fact about Harm Reduction is that much of the HR measures we can (and, I submit, should) take are measures that reduce the harm *that is a consequence of other, more punitive policies we have in place.* For example, overdose is a real and ever-present risk with many (but not all) illicit drugs in virtue of the fact that drugs are a black-market product sold in the absence of a regulatory framework: the strength of a quantity of any given drug can vary dramatically from purchase to purchase, and drugs are often adulterated with cheaper substances—which can themselves pose toxicity risks, or interact differently to the expected drug with other drugs like alcohol—in order to increase profits for sellers. It is certainly true that one could fatally overdose on an opiate, or cocaine (*inter alia*) even if drug supplies were regulated and quality-assured. But the fact that they are not means that drug-taking is dramatically more risky than it would otherwise be were drugs not illegal. Add to this a ‘just say no’ drugs education regime—in which education on safe dosing is not available—and the conditions are put perfectly in place for avoidable overdose.

Likewise, injection drug use poses the risk of contraction of HIV and other blood-borne viruses (BBV) through needle-sharing; whereas such risk is not usually associated with type-1 diabetes, sufferers of which routinely inject insulin. In the UK, this is because the s9A of the Misuse of Drugs Act 1971 prohibits the supply of

any article which may be used…in the administration by any person of a controlled drug to himself or another, believing that the article (or the article as adapted) is to be so used in circumstances where the administration is unlawful….

In other words, in the normal run of things, sale of clean needles to anyone *suspected* of intending their use for drug injection is a criminal offence. Most western jurisdictions have similar legislation. Needle exchange programmes simply (incompletely) undo the effects of these laws on the risk profile of drug injecting.

What this means is that dHR measures that reduce the risk of overdose and BBV infection—as well as many other risks—act to counteract *non*-*native* risks of drug use: in particular, risk that exists because of current policy, rather than because drugs ‘just are’ risky in that way. dHR, then, is sometimes making drugs safer than they would be, say, in a state of nature; but rather oftener dHR measures are simply *undoing risk we’ve created as a society* through punitive drug laws.

## Justice as Desert

Moral desert in broad brushstrokes is easily accessible and should be familiar to anyone who’s ever thought about how to treat others. When we say that *Amy deserves a gold star for hard work* or *Mary deserves to be excluded because she’s being mean*, we’re in moral desert territory. The basic idea is that what you’ve done (or who you are, or what’s been done to you, etc.) makes a difference to how we (or society, or fate, etc.) ought to treat you now.

Moral philosophers pick out two key features of moral desert. First, desert ‘must have a base’ [2]: there must be some action, or attribute, or biographical fact about the person in question that explains the desert. Philosophers these days usually agree that the desert base cannot be an inborn fact about one’s character—what Rawls calls a ‘native endowment’—for example, that Amy is naturally hard-working or that Mary is just vicious by nature—since it often seems like we have little control over such facts about our own characters, and it’s therefore unjust to reward or punish people for such facts (see [3, 9, 10]). Most of us, philosopher or otherwise, think of desert as something that is tied more or less to responsibility for past actions or events (cf. [3]).

Second, what we deserve is tied to or related to the basis for such desert. So for example, on some theories of moral desert, a particular past action always points to a particular desert: *Suzanne deserves overtime pay because she stayed late at work* or *Colin deserves a slice of cake since he helped make it* [9]. Alternately, some theories take desert as more of an aggregate concept, so that what you do in general points at what you deserve in general: *Robin deserves good friends since he’s always so thoughtful* [11]. Either way, there’s a correlation between the quality of your actions (desert base) and how others should treat you (desert).

Justice as moral desert is simply the idea that a just state of affairs is one in which everybody gets what they deserve: receipt according to desert is what justice *is*, on this understanding of justice. So, for the world to be right, on this conception, we not only need for Suzanne to receive money and Robin to have nice friends, we also need for Mary to be shunned. While it might be kind and charitable to treat Mary better than she treats others, it isn’t strictly speaking *just* to do so.

## dHR and Desert

My claim in this paper is that even on a desert account of justice—that is, even if we as a society think that what is fair and just is that everyone gets what they deserve, no more, no less—dHR is fair and just policy. PWUD deserve good health, care and concern in the same way that we all do, and for PWUD, given the context in which their use occurs, being deserving of good health means being deserving of dHR.

The broad thinking behind the idea that dHR flouts justice as desert, again, is the thought that, since PWUD create and maintain the circumstances that put them in harm’s way through their own bad actions, we are under no obligation to aid them in avoiding harm; and strict justice dictates, further, that we not do so, since what is fair and just is that everyone gets what they deserve. On close inspection, there are several problems with this line of thinking.

### Viciousness

First, since justice as desert has it that justice is *happiness according to virtue*, we would need to show that using drugs is antithetical to virtue, in order to show that the happiness granted to PWUD by measures that reduce the harms of drug use would be unjust. That is, we would need to show that drug use is vicious—morally wrong, bad—and thereby deserving of unhappiness. And we would need to be able to say why this is so *even setting aside the harms of drug use*, since dHR can and would change what harms attach to drug use. Many people do, indeed, feel that drug use is vicious (we use the word ‘vice’ to describe it in everyday speech) even setting aside harms; but one would be hard pressed to cite a principled reason for this view, one that would separate the would-be moral judgement from a mere cultural bias.

### Fittingness

Further, even if there is a principled reason to suppose that drug use is vicious, there is a poor fit between the act of using drugs, as desert base, and avoidable harm as desert for at least two reasons.

The first reason is that, while drug use is a finite act of (let’s suppose) vice—perhaps repeated throughout a life, but surrounded by other, morally-meaningful day to day activities—health and other harms associated with drug use can be life-long, measurably impacting overall wellbeing, and not just momentary unhappiness. ‘Drug use (desert base) deserves poor health and wellbeing (desert)’ is just not a fit for the ‘particular past action points to a particular desert’ mould articulated above, since any particular instance of drug use, even if we grant that it is vicious, is comparatively minor to any of the pantheon of vicious acts we might think deserve life-altering negative consequences (murder, rape, ecological destruction, and so on).

Neither is it a good fit for the idea that what you do in general points to what you deserve in general: most PWUD do not spend the majority of their time using drugs [6]. The majority of their time is spent doing the day to day things that all of us do: spending time with friends and family, working, sleeping, etc. So, a poor quality of life—a risk of death, even—is not a fitting desert for PWUD even if we take an in-general view of their actions, since the aggregate of all of a PWUDs actions in life taken together will not—all other things equal—generate desert so severe.

We might, in this case, worry that the person who uses drugs ‘heavily’ might still be deserving of avoidable bad consequences, or perhaps less deserving of dHR interventions if we take a ‘what you do in general’ approach to desert: after all, she may spend most of her time engaged in drug-taking or drug-related activity. If drug use is vicious, that seems like a pretty vicious life. It is also—in the case of drugs like heroin and cocaine—a pretty dangerous life, imbued with much more risk than that of occasional use might present. In reply to this worry, we might again point out that even if drug-taking is vicious, it is clearly not vicious on the order of, say, murder. So while we might feel that this ‘heavy’ PWUD deserves to suffer, it does not follow that she deserves death (even, I assume, for those of us who feel murderers deserve death). Declining to pursue dHR policies, or diminishing the level of intervention we offer, among other things, increases her risk of just that.

The second reason that ill health is a poor fit, desert-wise, for drug use has already been canvassed above: the harms of drug use are driven, in significant part, by decisions we make as a culture, decisions that are outside of the control of the individual who uses drugs. Unavailability of a safe drug supply, de facto prohibitions on the dissemination of information on safe dosing and use, lack of access to clean injection equipment, and so on, are all consequences of current drugs policy, rather than native to drug use: a little like throwing rocks at cyclists and then declaring cycling dangerous on grounds of rock-injury risk. Digging our heels in and declaring that ‘cyclists know the risk [of being hit by rocks that we are throwing] so they *deserve* the consequences’ is a deck-stacking that takes us well out of range of any reasonable claim to just desert.

One might object that whether desert ‘fits’ desert base, with respect to the scope of the would-be desert, is a philosopher’s worry: avoidable lack of wellbeing as a consequence of using drugs might not fit *the theory*, but it fits reality. But this seems implausible, since most of us are happy to suppose that desert should fit base in most every-day thinking. Most of us would not be happy, for example, with the biggest Christmas bonus going to the employee with the most colourful ties, even if we think it right that such an employee should be praised for his cheerful attire. So, the idea that desert needs to ‘fit’ desert base is not just a philosopher’s niggle. It is probably built into the notions of what is just and fair that most of us have.

### Rewarding Bad Behaviour

One might worry that, even given the poor fit between drug use and drug risks with respect to desert, there is something odious about rewarding PWUD for taking drugs by taking special care to see that they are safe and well when they use. We might think this even if there is no principled reason to suppose that their actions are vicious.

According to this worry, dHR is special treatment, like giving a child an extra scoop of ice cream or giving an employee a bonus. If the child is fussy, or the employee’s performance is distinctly ho-hum, rewarding them thusly seems imprudent, since we are not simply letting them ‘get away with it’, but are actually taking positive steps to improve on the natural outcome of their behaviour for them. We might be encouraging them to behave sub-optimally (if not downright badly) in future.^3^

A moment’s reflection shows this worry to be unfounded. First, as was said above, many of the risks associated with drug use are non-natural to the use. That is, many of the risks are generated by the social and policy decisions we make and are out of the control of the individual PWUD. Taking away those risks, then, does not represent taking positive steps to improve the risk profile. Rather, it represents a removal of risks placed on the shoulders of PWUD by our own collective decisions as a culture: more like removing the asbestos from the employee’s workplace than like giving the employee a bonus.

Second, even those risks that are native to drug use (i.e. not generated by policy decisions like restrictions on the purchase of sterile injection equipment) are such that removal of them does not represent a reward for the behaviour. Compare with seatbelts. Seatbelts reduce the native risk of riding in a car. They are not a benefit or special prize for people who travel by car, and nor do they encourage people to travel by car. Seatbelts are perfectly compatible with, for example, public campaigns to reduce car travel.

### Measuring Desert

Even if we suppose that drug use is vicious, it isn’t clear how use should factor into our assessment of what an individual deserves, and it isn’t clear what it would imply about what they deserve, nor how strongly.

Just as many of the risks associated with drug use are dependent on facts about community and state attitudes and policy, whether a person uses drugs problematically seems to be heavily influenced by context—socioeconomic context, peer group and living standards, etc. [13]. One study, for example, found that ‘neighbourhood disadvantage was a significant predictor of frequency of injection drug use’ [5]. If this is right, then it is unclear where the individual decision ends and outside forces begin; and if that is so, it isn’t clear that what PWUD deserve can be straightforwardly gleaned from their decisions to use drugs, since that decision will not be their free will and responsibility alone but will be driven to some extent by outside forces. This means that the act will not, in some sense, be wholly desert-relevant, since we tend to think that what one deserves depends on agent-centric facts about the individual and her beahaviour: on the things she does for which she bears moral responsibility, rather than on the things that are done *to her*.

Setting aside worries over individual responsibility for drug use, it is still unclear what desert drug use would imply. There are two interrelated theoretical issues around justice as desert that bear importantly on this question. First, in order to get a handle on what people deserve, we need to determine what constitutes default desert. That is, what’s the starting point for just treatment, such that good or bad actions can take someone above or below that baseline of desert—how ought we treat others in the absence of virtuous or vicious behaviour, or more plausibly, in cases where another’s actions overall are virtuous and vicious in equal measure (cf. [11])?

The obvious answer to this question might be: neutrally. It might be that we ought to treat a morally neutral person neither well nor badly. But it’s hard to get a fix on what it would mean to treat a person neutrally. It’s not even clear that there is such a thing. Further, most people pretheoretically believe that, all other things equal, we ought to be *kind*. If this common-sense preference for kindness is right, it stands to reason that the sort of kindness we should show to a morally neutral person will be a mild, minimally-kind sort, since while we believe we should treat others with kindness, we also tend to hold that there is plenty of room for treatment of others that goes ‘above and beyond’. That is, the baseline must fall short of the full measure of kindness we *could* show to others; or perhaps, that we *should* show to others if their actions warrant it.

If this is right—if minimal kindness is the default desert—then what PWUD deserve, assuming we cannot give a principled reason for holding that drug use is vicious, is kindness. If we can give a principled reason for holding that drug use is vicious, then PWUD may deserve something short of kindness, which may or may not sink to the level of cruelty, depending on the strength of the viciousness of drug use. But even if it does sink to the level of cruelty, there’s cruelty and then there’s cruelty. Just as we would be loath to accept a punch in the face for rudeness, we ought not to accept—indeed, contribute to—avoidable death or serious injury for drug use.

If we reject the notion that everyone deserves minimally kind treatment in the absence of vicious or virtuous action, we have other familiar concepts to draw on in order to understand baseline or background desert. Importantly, human rights can be seen to function as articulations of minimally morally acceptable treatment of other persons, regardless of desert: they are what simply being a human person entitles one to. We can take them, then, as recognised and accepted articulations of background desert, albeit a minimal conception below which virtually no bad action can generate desert. In the various declarations on human rights in force today, certain themes are prevalent and salient to the dHR question, such as health, security of the person, life, well-being (UDHR, ECHR, etc.). None of these basic rights is consistent with knowingly increasing the suffering of PWUD via a preference for punitive policymaking. Taking action to minimise the avoidable harms to PWUD by implementing dHR measures in the face of punitive drug policies is the very minimum action consistent with desert, where human rights are understood to articulate a baseline desert position, even if drug use is vicious.

Both minimal kindness and human rights are at least as well motivated—qua articulations of baseline desert—as is desert as an articulation of justice. That is, if it stands to reason that people ought to get what they deserve, then it stands to reason that either kindness or human rights are what everyone deserves, all other things equal.

The second theoretical question concerns the strength or duration of our obligation to others in the face of a given desert—particularly where we take desert to be aggregate and/or scalar. If someone deserves happiness because they are virtuous, what obligation does that generate for others? If we act so as to provide *some* happiness to such a person, but not as very much happiness as we might do, have we fulfilled our obligation to them? Have they got what they deserve from us, as individual moral agents? Or is justice only served, as it were, if they are just as happy as they deserve to be?

This question bears importantly on the claim that PWUD deserve dHR, if their desert is grounded in (something like) a basic right to good health. If PWUD deserve dHR in virtue of deserving good health, one might wonder why PWUD deserve dHR, rather than effort towards drug use cessation. It stands to reason that one’s health would be better—and more likely to be good—were one not to use drugs at all. Why, then, should we implement dHR policies rather than abstinence policies?

The first and most important way to reply to this worry, of course, is to point out that there is no *pro tanto* conflict between dHR and cessation assistance: done thoughtfully, we can both implement policies that reduce harm to PWUD and provide pathways to abstinence. Just as seatbelts don’t stop anyone taking up cycling—or a community advocating for reduced car use—dHR does not stand in the way of measures to help people kick problematic drug habits.

Secondly, we might point out that most PWUD use drugs non-problematically: The Global Commission on Drug Policy 2019 Report, for example, estimates that around 11% of PWUD exhibit problematic use. In other words, for the lion’s share of PWUD, there’s a good chance that the *native* effects of their drug use are simply not significantly impacting on their health—perhaps no more so than other unhealthy habits, like eating ‘junk food’ or failing to take regular exercise.

## Conclusion

Even on a desert conception of justice, and even if there is principled reason to suppose that drug use is vicious, dHR policies and practices align with justice. Even if we accept the claim that drug use is morally wrong, rather than simply socially unacceptable, refraining from taking steps to minimise the harms of drug use for PWUD is out of step with justice, in that much of the harm PWUD suffer is outside the control of the drug user, but largely within the control of the society that generates drugs policy, and is disproportionate to the act of using drugs. Further, just as seatbelts do not encourage car use, dHR need not encourage drug-taking: it can simply reduce risk. Finally, I have argued that either kindness or basic rights should characterise our conception of baseline moral desert, such that even if drug use is vicious, dHR is the minimum that PWUD deserve. If our aim is to craft policies that foster justice, drug policy must seek to reduce the harms suffered by people who use drugs.^4^

## Footnotes

1 There are, of course, other and often similar grounds on which one might oppose HR. Importantly: one might suppose that we are all responsible for the risks we take on, full stop. So, moral desert might be neither here nor there: you knew the risks, you’re responsible for them. A full treatment of such an argument is out of the scope of this essay, but as I will argue below, such an argument would not be successful against dHR since many of the risks of drug use are risks generated by policy. This means that we (policymakers and community members who support the policy) cannot wash our hands of those risks. In this paper I’ll be focussing just on moral-desert-based opposition to dHR.

2 Some dHR measures are designed primarily or partially to benefit *society*, rather than the individual PWUD. For example, many needle exchange programmes are just that: *exchange* programmes, designed to bring in old needles—keep them off the streets—as much as to provide new ones. Justification for socially-beneficial (rather than directly beneficial to the PWUDs) measures will obviously not be dependent on what PWUDs themselves deserve. I’ll focus, in this paper, on whether we can justify benefitting PWUDs themselves on desert grounds.

3 Indeed, we may encourage PWUDs to engage in even riskier drug-taking. Whether this outcome does or would occur is an empirical matter rather than a theoretical one—factual rather than evaluative. If it is the case that PWUDs take more risks when dHR measures are in place, then the harm resultant from the increased risk should be factored into the desert assessment.

4 I would like to thank John Coggon, participants in the Harm Reduction panel at the 2019 Canadian Philosophical Association Annual Meeting, and two anonymous reviewers for invaluable feedback on this work.
